# Driver of discontent or escape vehicle: the affective consequences of mindwandering

**DOI:** 10.3389/fpsyg.2013.00477

**Published:** 2013-07-25

**Authors:** Malia F. Mason, Kevin Brown, Raymond A. Mar, Jonathan Smallwood

**Affiliations:** ^1^Management Division, Columbia UniversityNew York, NY, USA; ^2^Department of Chemical and Biomolecular Engineering, University of ConnecticutStorrs, CT, USA; ^3^Department of Marine Sciences, University of ConnecticutGroton, CT, USA; ^4^Psychology Department, York UniversityToronto, ON, Canada; ^5^Department of Social Neuroscience, Max Plank Institute for Human Cognitive and Brain SciencesLeipzig, Germany

**Keywords:** mindwandering, attention, wellbeing, default network, affect

## Abstract

An emerging body of evidence suggests that our penchant for entertaining thoughts that are unrelated to ongoing activities might be a detriment to our emotional wellbeing. In light of this evidence, researchers have posited that mindwandering is a cause rather than a manifestation of discontent. We review the evidence in support of this viewpoint. We then consider this evidence in a broader context—with regards to mindwandering's antecedents, respecting the observation that people frequently find pleasure in their off-task moments, and in light of the lay beliefs people hold about its causes. We report data from two studies that speak to the potential challenges of establishing a definitive causal link between mindwandering and wellbeing. First, to advance the idea that mindwandering can convey affective benefits, in spite of negative feelings about mental disengagement, we examined cortical responses in a unique individual who presents with a long history of excessive—but enjoyable—task-irrelevant thinking. Second, to explore the idea that lay beliefs about mindwandering may substantially color the affective responses people have to a mindwandering episode, we surveyed people's beliefs about mindwandering's antecedents and related them to the affective reactions people anticipated to off-task moments. Our hope is to provide a nuanced evaluation of the available evidence for the assertion that mindwandering causes unhappiness, and to provide a clear direction forward to better evaluate this possibility.

People spend an estimated one third to one half of their waking lives mindwandering (Kane et al., 2007; Killingsworth and Gilbert, 2010). The sheer ubiquity of these off-task moments has prompted researchers to consider how mindwandering might impact our emotional lives. How does frequently entertaining task-irrelevant thoughts make us feel? An emerging body of evidence—including our own (e.g., Smallwood et al., 2007, 2009a; Mar et al., 2012)—indicates that mindwandering can be associated with diminished psychological well-being. These findings and others have prompted some researchers to conclude that mindwandering causes unhappiness (e.g., Killingsworth and Gilbert, 2010). Although we certainly agree mindwandering can be a source of discontent, we suggest its relationship with well-being may be complex. In this piece, we discuss the barriers to drawing a causal link between attentional lapses and diminished well-being. We also review and attempt to reconcile the conflicting evidence surrounding the relation between mindwandering and emotional states. In doing so, our hope is to provide a nuanced evaluation of the available evidence for the assertion that mindwandering causes unhappiness, and to provide a clear direction forward to better evaluate this possibility.

We begin by summarizing existing evidence in support of the view that mindwandering causes unhappiness. We then consider this evidence in light of two factors: (1) the known triggers of mindwandering and (2) the mental states of individuals who frequently mindwander. We then attempt to reconcile evidence that mindwandering causes discontent with evidence that people frequently find pleasure in their off-task moments, arguing for a distinction between affect experienced during mindwandering and affective reactions to noting one's mind has strayed. We close with a discussion of how lay beliefs about why the mind wanders might shape people's perceptions of their moods following a mindwandering episode.

## Evidence of a relationship between mindwandering and negative affect

A small but burgeoning body of evidence suggests that negative affect tends to accompany failure to attend to an ongoing task. For example, frequent absentmindedness is associated with lower affective well-being, based on self-reports of the propensity to experience brief lapses of attention (Cheyne et al., 2006). Follow-up work clarified that a chronic inability to engage in and sustain attention toward an ongoing task frequently gives rise to boredom, which in turn results in persistent negative affect (Carriere et al., 2008). Self-reported frequency of mindwandering has also been related to negative affect in both North America and Europe (Mar et al., 2012; Stawarczyk et al., 2012). Consistent with these findings, people who experience more frequent dysphoric episodes also appear to report more frequent absentmindedness and exhibit a diminished capacity for sustained attention (Watts et al., 1988; Wagle et al., 1999; Farrin et al., 2003). Smallwood and colleagues have documented a positive correlation between dysphoria and frequent mindwandering across a wide range of cognitive tasks, including word learning (Smallwood et al., 2003, 2004b), sustained attention (Smallwood et al., 2004a), and simple word-fragment completion (Smallwood et al., 2004b).

In light of these associations, a key challenge researchers confront moving forward is determining the directionality of any causal relationship that might exist between mindwandering and negative affect. Although our focus is the extent to which there is evidence that mindwandering causes diminished well-being, it is important to point out that a case has already been made for the reverse: negative affect causing more frequent mindwandering. For example, Smallwood and colleagues argue that negative moods diminish the amount of attention people commit to an ongoing task, giving way to task-irrelevant thoughts regarding personal concerns (Smallwood et al., 2005, 2007, 2009a,b see also Power and Dalgleish, 1997; Teasdale, 1999) and past events (Smallwood and O'Connor, 2011). In line with this argument, negative affect is especially likely to impair sustained attention when people have highly accessible, unresolved personal concerns (Lyubomirsky et al., 2003). It is worth noting that the argument that negative mood increases off-task thinking is based largely on data that is correlational and thus insufficient for making definitive causal claims. Does compelling evidence of the reverse causal relationship exist: How much confidence should we place in the claim that mindwandering directly diminishes feelings of well-being?

In recent work, Marchetti et al. (2012) examined whether mindwandering diminishes well-being and if this is a result of people (1) entertaining negative thoughts during mindwandering episodes or (2) mindwandering increasing self-focus which in turn leads to dysphoria (Mor and Winquist, 2002). Contrary to the view that the effect of mindwandering on well-being is mediated by negative thinking, the authors found no evidence that people who experience more frequent mindwandering entertain more negative thoughts in off task-moments. Nor did they find a relationship between negative thinking while mindwandering and diminished feelings of well-being. They did, however, report finding that a diminished ability to sustain attention on a current task is associated with heightened accessibility of negative constructs (as measured with a scrambled sentence task involving negative words) among people with moderate to high levels of depressive symptoms. The authors interpret this as evidence that fully coupling attention to an external task prevents people who are predisposed to negativity or rumination from entertaining disturbing thoughts. Although these results are interesting, they do not provide strong evidence that mindwandering causes negative affect because (1) off-task thinking related to an increase in the accessibility of negative constructs only among those who were already depressed (and even then, not causally so) and (2) there was no evidence that more frequent mindwandering among people with moderate to high levels of depressive symptoms was associated with heightened experience of negative feelings.

The view that decoupling attention from events in the here and now diminishes well-being is common among researchers who study mindfulness, with mindfulness defined as “the state of being attentive to and aware of what is taking place in the present” (Brown and Ryan, 2003; p.822; for review see Brown et al., 2007; Keng et al., 2011). There is evidence in favor of this view from mindfulness research, with people low in trait mindfulness also reporting a greater number of depressive symptoms, more unpleasant affect, lower life satisfaction, and a greater number of recent visits to medical professionals than those high in mindfulness (Brown and Ryan, 2003, Study 1; Carlson and Brown, 2005). Low levels of trait mindfulness are also associated with a tendency toward rumination (Raes and Williams, 2010), difficulties in emotion regulation (Baer et al., 2006), as well as more frequent negative spontaneous thoughts and a diminished ability to disregard those thoughts (Frewen et al., 2008). These studies are correlational in nature, and do not support causal inferences, however. Consistent with the view that momentary fluctuations in mindfulness prompt changes in affect, results of experience-sampling studies reveal that when individuals are less attentive to the day-to-day activities in which they are engaged, they also report more unpleasant emotion and diminished feelings of control (Brown and Ryan, 2003, Study 4; see also Killingsworth and Gilbert, 2010).

Aside from the correlational studies, there is work demonstrating that learning to be mindful of ongoing activities is followed by improvements in well-being. In two landmark intervention studies, Kabta-Zinn and colleagues found that enrolling in a standardized 8-week mindfulness program was associated with diminished unpleasant symptoms among patients with chronic pain (Kabat-Zinn et al., 1985) and anxiety (Kabat-Zinn et al., 1992). Numerous researchers have since demonstrated that mindfulness training reduces the risk of relapse into depression and diminishes other depression-related outcomes (cf., Teasdale et al., 2000; Ma and Teasdale, 2004; Bondolfi et al., 2010; Godfrin and Van Heeringen, 2010; for recent review see Chiesa and Serretti, 2011). Although one can make a stronger case for a causal effect of attention on well-being based on this evidence, note that these studies generally only test whether compliance with the training is associated with subsequent improvements in well-being; this is not the same as showing that increases in present-oriented attention mediate the improvements in well-being (see, however, Brown and Ryan, 2003, Study 5).

It is also worth mentioning recent evidence that mindwandering plays a role in creative problem-solving. Compared to rest or engaging tasks, tasks that promote mindwandering seem to facilitate divergent thinking (Baird et al., 2012; see also Sio and Ormerod, 2009). Although the precise mechanism by which mindwandering enhances creativity is uncertain, a large body of research reveals that positive mood enhances divergent thinking (e.g., Abele-Brehm, 1992; Baas et al., 2008). Thus, it is possible that mindwandering enhances creativity by improving mood.

Perhaps the strongest evidence for the view that mindwandering causes psychological distress is found in the work of Killingsworth and Gilbert (2010). These authors developed a smartphone application to sample the thoughts and feelings of 2250 adult participants at random intervals. A few times each day, participants were prompted to indicate their level of happiness, report the activity in which they were currently engaged, and judge whether their mind was anchored on this activity (i.e., whether they were task-engaged or mindwandering). People were happier when their attention was directed at what they were doing compared to when their attention had wandered to task-irrelevant information. Furthermore, they observed that previous mindwandering predicted later unhappiness, but that current unhappiness did not predict subsequent mindwandering. They thus concluded that mindwandering is the cause, and not the consequence, of unhappiness. This work is impressive for a number of reasons, including the sample size, the ecologically-valid context, and the daily diary approach. However, their conclusion—that mindwandering causes unhappiness—should be examined more closely for a number of reasons, in our opinion. For one, the researchers randomly sampled their participants a maximum of three times a day. Thus the time-series approach they adopt to establish causality involves hours-long lags between current unhappiness and previous and subsequent mindwandering. The longitudinal samples may be spaced too far apart (several hours) to be credible for causal analysis. In the next section, we consider another potential problem that is common in mindwandering research: unaccounted-for third variables.

## Accounting for the effect of “third variables”

An obvious issue with correlational data is that inferring the direction of causality is difficult. Does mindwandering diminish well-being or does diminished well-being trigger mindwandering? Or are both possibilities true? These questions cannot be answered unequivocally with purely correlational data. A second potential issue, one we feel is easy to overlook, is that unobserved third factors might be responsible for the correlation between mindwandering and well-being. For example, dispositional anxiety or situational stress can make focusing on a given task difficult, and both are also directly related to decreased well-being. Unobserved variables could account for a mindwandering-wellbeing association, even if the data have a temporal component, as is the case with the time-lagged analysis performed by Killingsworth and Gilbert (2010). This problem of spurious correlation, in which two variables are correlated but not due to any direct causal relationship, is an issue closely related to the omitted variable bias in regression analyses (Greene, 2002; Clarke, 2005). A credible causal argument about the emotional consequences of mindwandering must involve a genuine effort to measure and account for third variables that might cause both discontent and mindwandering. This is bound to be difficult, especially in the absence of a fully formed component process model that can help identify what processes control the initiation of a mindwandering episode (Smallwood, 2013).

Researchers seeking definitive evidence that mindwandering causes discontent would be well-served by not only gathering data that make directional claims possible, but also by attempting to rule out competing explanations when collecting correlational data. We would like to stress that both approaches are valuable. Although experimental manipulations allow for causal inferences, this approach suffers from reduced ecological validity. While cross-sectional survey data introduces problems with properly inferring causal direction, they allow us to examine mindwandering in a more ecologically valid context. In the case of mindwandering and well-being, there are a host of possible candidate third variables that are important to consider, including task characteristics, life events, and individual dispositions. We highlight some of them here.

### Task characteristics

#### Invariability/slow pace

Monotonous, invariable events that are slow to progress are known to both prompt an increase in mindwandering (Antrobus et al., 1966) and to diminish well-being (Neu, 1998). Unsurprisingly, boring tasks encourage mindwandering and diminish wellbeing. Hence, in research examining the relation between mindwandering and negative affect, probing for the concomitant presence of monotonous circumstances that could cause an increase in both is therefore important.

### Life events

#### Unresolved personal concerns

According to the current concerns theory (Klinger, 1971, 1977, 1996), committing to any goal potentiates emotional responses to and cognitive processing of internal and external cues associated with its pursuit. As a consequence, between committing to a goal and attaining it, people are more likely to spontaneously think about the goal than they would have otherwise. Consistent with this motivational account of thought content, a large and burgeoning body of evidence suggests people often reflect on unfulfilled goals while mindwandering (e.g., Klinger, 1977; Klinger et al., 1980; Mason et al., 2009), especially goals whose attainment has become problematic (Klinger, 1977; Klinger et al., 1980). Thus, doubt about one's ability to attain an important, unfulfilled goal can increase mindwandering and can also diminish well-being, possibly creating a spurious relationship between the two.

#### Disconcerting news

Another possible third variable related to unresolved personal concerns is the introduction of disconcerting news. Receiving disconcerting news can cause both increased mindwandering and diminished well-being. For example, in their seminal work, Antrobus et al. (1966) exposed participants to a radio broadcast announcing that China was entering the Vietnam War and the United States was planning a retaliation. This cover story was credible given that the study was conducted in June of 1965, during the initial escalation of the Vietnam War. Participants exposed to this broadcast reported a significant increase in task-irrelevant thinking and also experienced stronger feelings of despair, worry, anxiety, fear, and so forth. In fact, the work of Horowitz (1975) suggests that the disconcerting event need only be moderately stressful to cause both an increase in off-task thought and a decrease in well-being. Experimental studies that involve a stressful event must therefore establish that any association observed between mindwandering frequency and negative affect is not due to the independent effect of receiving disconcerting news or of being placed under stress. For correlational data, measuring and controlling for situational stress is similarly important.

### Individual dispositions

#### Depression

As discussed above, a large body of evidence reveals that depressed individuals exhibit higher levels of mindwandering than their non-depressed counterparts (Watts et al., 1988; Smallwood et al., 2007). Levels of depressive symptoms in sub-clinical participant populations also predict mindwandering frequency (Smallwood et al., 2003, 2005). This state of affairs raises the possibility that mindwandering does not create dysphoria, but rather that both are a consequence of a common cause: depression. The view that heightened mindwandering and diminished affect might co-occur because they are both independently related to depression is consistent with the findings of Marchetti et al. (2012), who only observe the link between mindwandering and negative cognitions in those with moderate to high levels of depression.

#### Neuroticism

Evidence suggests that neuroticism—a personality trait characterized by anxiety, moodiness, worry, and envy—might also explain the relationship between mindwandering and dysphoria. Compared to their low-neuroticism counterparts, people high on this trait experience more frequent mindwandering (e.g., Baer et al., 2006; Giluk, 2009) and report lower levels of well-being (e.g., Diener et al., 1999). Correlations between mindwandering and well-being might reflect the fact that neuroticism prompts more off-task thinking and has a separate, diminishing effect on well-being. For example, Brown and Ryan (2003) reported that the tendency for people who were low in “present-oriented thinking” (i.e., trait mindfulness) to report diminished levels of well-being reduced dramatically when the authors controlled for the effect of neuroticism.

#### Mental self-regulation/trait mindfulness

A third individual difference that might give rise to a spurious association between mindwandering and negative affect is mental self-regulation. People who are poor at regulating their mental contents can be thought of as being high in the trait tendency toward mindwandering. These individuals experience more frequent mindwandering (Brown and Ryan, 2003; Burg and Michalak, 2011; Mrazek et al., 2012) and are less effective at shaping both the affective reactions they have to their thoughts (e.g., reframing negative reactions to internal and external events, etc.; Frewen et al., 2008; Keng et al., 2011) and correcting/repairing unpleasant mood states (Brown and Ryan, 2003; Baer et al., 2004; Creswell et al., 2007). These findings raise the possibility that mindwandering does not cause dysphoria, but rather both are a consequence of poor mental self-regulation. Consistent with this possibility, a recent study found that trait mindfulness fully explains the relationship between mindwandering frequency and psychological distress (Stawarczyk et al., 2012; however, see Brown and Ryan, 2003, Study 5). This finding, and others, illustrates the importance of measuring and controlling for possible third variables. In this study, mindwandering did not directly cause discontent in the sample of participants; rather, both were a consequence of poor mental self-regulation.

Although certainly not exhaustive, this list of possible third variables that could account for relations between mindwandering and dysphoria serves an illustrative purpose. Absent measurement and control of the individual dispositions, life events, and task characteristics that cause changes in both mindwandering and mood, identifying whether mindwandering causally impacts happiness or if the two are spuriously correlated is difficult. The list helps to demonstrate that assertions regarding causality are greatly undermined when possibly confounding factors are not measured and taken into account. For example, observing a link between mindwandering and diminished well-being becomes more compelling when these variables are measured and taken into account. More generally, to move beyond this descriptive analysis, it will be necessary to develop a fully specified model of the mind-wandering state from which clear and unambiguous predictions can be made regarding the hypothesized mechanism by which mind-wandering influences mood (and vice-versa).

## Affective reactions to mindwandering versus affective experiences while mindwandering

Beyond possible third variables that might cause both mindwandering and negative affect, another barrier to evaluating the veracity of causal claims is a precise understanding of what such an account predicts. For example, it is not entirely clear if the argument being made is that the more one mindwanders on average, the greater one's average level of unhappiness or if the claim is that mindwandering has an immediate, transitory effect on mometary mood states. Perhaps both are true? Not only is there uncertainty around the timeframe and duration of mindwandering's effects, the nature and source of the negativity are unclear. One view is that mindwandering diminishes wellbeing because the experience itself is inherently unpleasant. Alternatively, the diminished well-being might reflect the fact that people respond to their mental meandering with frustration and concern over the lack of control they have over their attention. Our point is that judging the strength and coherence of the available evidence is challenging when the accounts of mindwandering are underspecified.

We propose that one step toward resolving this issue involves drawing a distinction between the affect experienced during mindwandering and affective reactions to noticing one's mind has strayed. It seems possible for people to derive pleasure from a mindwandering act but still react negatively to noticing that their attention has drifted to task-unrelated matters. By respecting the difference between the two, we might reconcile some of the inconsistencies in the current corpus of evidence.

To this point, we have focused on evidence that mindwandering is associated with diminished well-being and have ignored the affective benefits of decoupling ourselves from the current sensory environment. Contrary to the view that sustained-attention failures necessarily lead to diminished well-being is a body of evidence showing that people allow their minds to wander to cope with dull or stressful task settings. For example, people commonly report using daydreams to ease boredom at work (Singer, 1961; Molstad, 1986; Fisher, 1987). Individuals with a proclivity for mindwandering also exhibit less physiological reactance to stressful events (Singer and Antrobus, 1963) and asking people to mentally wander from the current moment diminishes the stress response they exhibit in anticipation of an electrical shock (Rowe, 1963). In children, a strong proclivity toward mindwandering is associated with enhanced patience and self-control (Singer, 1961). In other words, ample evidence suggests people look to mindwandering to provide emotional respite from stressful or boring circumstances. To the extent that people are drawn to entertain themselves with internal reveries, they depend less on the external world for entertainment.

Could people enjoy the mindwandering experience but feel negatively about their mental disengagement? An initial evaluation of this proposal is available. We examined cortical responses in a unique individual recently described by Schupak and Rosenthal (2009). This person presents with a long history of uncontrolled and excessive daydreaming that is not coincident with any other clinical disorders. This individual reports concern about the amount of her daydreaming, yet considers it a “treasured activity.” We examined the neural correlates of her mindwandering, using functional magnetic resonance imaging (fMRI), and undertook two studies: (1) we contrasted mindwandering with both task-focused thought and periods of mindwandering suppression, and (2) we observed intrinsic correlations in brain activity during unconstrained thought (i.e., seed-based resting-state functional connectivity analysis; see Appendix for details).

We were interested in determining whether mindwandering is associated with increased fMRI responses in brain areas associated with positive, rewarding experiences. Brain imaging and electrophysiological work with non-human primates has identified the dorsal and ventral striatum, especially the nucleus accumbens (nACC), as important for the subjective experience of pleasure (e.g., Apicella et al., 1991). If mindwandering can be enjoyable or rewarding in someone who is chronically concerned about the frequency of its occurrence, activity in these areas should increase during periods of active and absorbing thought that is decoupled from the sensory environment.

**Figure 1:**
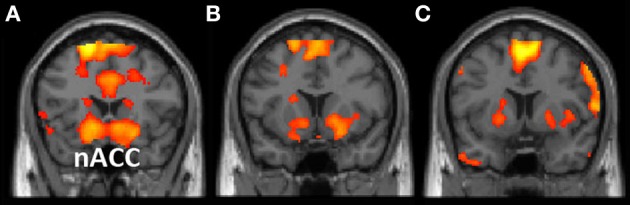
**(A,B)** Results of the “mindwander > concentrate” contrast, *p* < 0.005; *k* = 10. **(C)** Results of the “mindwander > suppress” contrast, *p* < 0.005; *k* = 10 superimposed. Results are displayed on the MNI single-subject TI anatomical image. nACC, nucleus accumbens.

**Figure 2:**
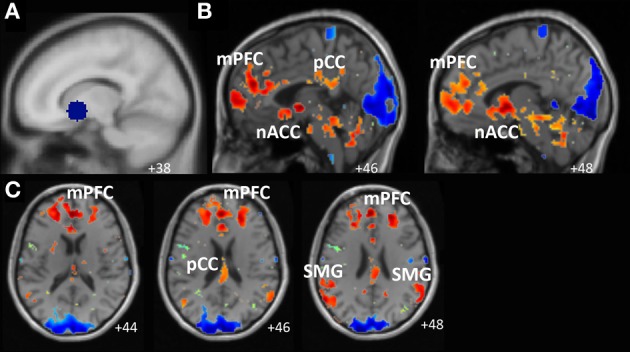
**Results of resting-state scan analysis revealed that the signal in the patient's nucleus accumbens (nACC) fluctuated with four default network regions: the medial prefrontal cortex (mPFC), the posterior cingulate cortex (pCC), and bilateral supramarginal gyri (SMG)**. Panel **(A)** depicts the seed regions used to generate the resting-state maps. Panel **(B)** depicts results from a sagittal orientation. Panel **(C)** depicts results from an axial orientation. Results displayed on participant's T1 anatomical image.

Consistent with this view, relative to periods of task-focused thought and mindwandering suppression, periods of active mindwandering were associated with increased activity in dorsal and ventral striatal regions, including the nACC (a reward area; Figure 1). Furthermore, our resting-state analysis revealed that signal in the nACC fluctuated with four regions associated with mindwandering [i.e., the default network (Mason et al., 2007; Christoff et al., 2009)]: the medial prefrontal cortex, the posterior cingulate cortex, and the bilateral supramarginal gyri (Figure 2).

Therefore, even in someone who is disturbed by a recalcitrant mind that will not “stay put,” the experience of mindwandering can be both subjectively pleasurable and biologically rewarding. These findings provide tentative support for the hypothesis that mindwandering can be a self-rewarding activity, even in the presence of concern regarding its frequency. By no means do we intend to suggest that people derive pleasure from all of their mindwandering experiences. We simply wish to highlight that the affect experienced while mindwandering and affective reactions to mindwandering are dissociable.

The core issue is that mindwandering seems to be a vehicle by which people project themselves from the current situations to which they are confined. The emotional consequences of this mental teleportation presumably depend on where it leads and if it interferes with an ongoing task to which one is committed. We have no doubt that mindwandering is a source of discontent and irritation, especially when it interferes with efficient and effective performance of a crucial current task. It also seems reasonable to assume that the emotional consequences of mindwandering depend on where the mind has wandered (cf., Mar et al., 2012). We suspect that mindwandering that involves a worrisome or negative topic will lead to diminished well-being, especially among individuals who are chronically distracted by pessimistic topics (e.g., individuals with depression). Consistent with this possibility, the thought “recycling” (i.e., rumination) that characterizes chronic depression is believed to increase both the length and severity of depressive episodes (e.g., Nolen-Hoeksema and Morrow, 1993).

Our point is that extrapolating beyond contrived experimental conditions to other contexts requires a clear and precise mechanistic story of the everyday emotional consequences of mindwandering. A complete mechanistic account would seem to require consideration of the content of off-task thoughts (Watkins, 2008, 2010; Critcher and Gilovich, 2010; Mar et al., 2012; Smallwood, 2013), the nature of the task from which the mind wandered, and whether the primary task requires full attention or if its simplicity allows people to entertain task-irrelevant thoughts concurrent to its pursuit (Teasdale et al., 1995; Mason et al., 2007; Smallwood et al., 2009b, 2011).

## Role of causal lay theories of mindwandering

In conjunction with the problem of third variables causing spurious correlations and distinguishing affective reactions to mindwandering from affective experiences while mindwandering, it is important to examine lay beliefs about mindwandering and how these might influence affective reactions to mindwandering. For decades, psychologists have noted that people construct plausible, quasi-scientific stories to understand, predict, and justify events in the world (e.g., Bruner, 1957; Heider, 1958; Kruglanski, 1980; Snyder and Gangestad, 1981; Hastie, 1983). These stories are known as implicit theories, lay theories, naïve theories, or causal schemata. Using these lay theories, people extrapolate from available data, construct explanations about what caused a certain event, and draw various inferences about themselves or others in relation to the event (Dweck et al., 1995; Morris et al., 2001). Some lay theories oversimplify the truth, whereas others are demonstrably wrong. However, all lay theories shape how people understand the phenomenon they are purported to describe. For example, despite having considerable experience observing tennis balls, water balloons, and other projectiles in flight, people have striking misconceptions about the principles that govern the trajectories of moving objects. Their mistakes are not random, but appear to arise from a widely shared intuitive theory of motion that adequately guides people's everyday interactions with moving objects but contradicts the laws of Newtonian mechanics (see Chi et al., 1981; McCloskey, 1983; DiSessa, 1993). These lay theories extend beyond the physical world to our shared social world, applied to such topics as trait personality (Kelly, 1955), intelligence (Dweck and Leggett, 1988), group behavior (Lickel et al., 2001), agency (Heider, 1958; Morris and Peng, 1994), and minds (Gopnik and Meltzoff, 1987; Wellman, 1990; cf. Ames and Mason, 2012).

With respect to lay beliefs about mindwandering, Critcher and Gilovich (2010) argue that mindwandering poses an attributional dilemma because an off-task moment can either be seen as resulting from the banal nature of an ongoing task or from some compelling property of the object of one's wandering mind. Either could prompt an off-task moment. Their results revealed that when the cause of mindwandering was ambiguous, participants resolved this atttributional dilemma by referencing the content of their mindwandering. Participants reported being bored by the task only when their mind wandered (1) to future or ongoing activities and not events of the past and (2) to multiple topics and not just one topic. They thus conclude that people forestall the deduction that their mindwandering is driven by task boredom when their musings involve an enjoyable event of the past or revolve around a single, distinctive topic versus several different ones.

These findings raise the possibility that people might similarly rely on a lay theory of mindwandering to understand how their off-task moments relate to their current well-being. That is, upon noticing one's mind has wandered from a current task, people might use their intuitive beliefs about when and why the mind wanders to infer a cause for the off-task moment and draw inferences about their current emotional state. Do people have beliefs about what causes their mindwandering? Is it possible that the feelings people report subsequent to an off-task moment depend on assumptions they make about its causes? It seems reasonable to expect that people who assume mindwandering is caused by personal flaws, impoverished activities, or disconcerting topics might respond to off-task moments with more distress than people who assume mindwandering occurs because attention tends to wax and wane naturally.

We obtained some preliminary evidence for this possibility by asking a sample of 361 participants (details in Appendix) about their affective reactions to and lay beliefs regarding mindwandering. More specifically, using the PANAS (Tellegen et al., 1988), we asked participants to indicate the extent to which they would feel various moods after a hypothetical mindwandering event. We also asked them to indicate the extent to which they agreed with a series of statements concerning the causes of mindwandering. As expected, participants commonly reported believing that dull tasks and distractible dispositions cause mindwandering. The belief that mindwandering occurs when important, enjoyable, or worrisome topics compete for attention to an ongoing tasks was also fairly common. Finally, there was some consensus around the idea that mindwandering is a natural feature of an information processing system that periodically disengages from ongoing events and activities (Table 1).

**Table 1:** **Lay theories regarding causes of mindwandering**.

**Cause**	**Mean**	***SD***	***t*-score**	**Mean diff.**	**Lower**	**Upper**
Occurs because I'm a distractible person	3.25	0.97	4.96^**^	0.25	0.15	0.35
Is caused by dull activities	3.85	0.80	20.07^**^	0.85	0.76	0.93
Is the result of my attention waxing and waning naturally	3.67	0.79	16.14^**^	0.67	0.59	0.75
Occurs because I enjoy thinking about the topics to which my mind wanders	3.41	0.80	9.89^**^	0.41	0.33	0.50
Occurs because the topics to which my mind wanders are important	3.34	0.79	8.19^**^	0.34	0.26	0.42
Occurs because the topics to which my mind wanders are worrisome	3.10	0.85	2.30^*^	0.10	0.01	0.19

Responses made using a Likert scale, ranging from 1 (Not at all true) to 5 (Definitely true) To determine if there was consensus around these beliefs we conducted a one-sample T-test (df = 360), examining whether the mean differed from the midpoint (3).

* p < 0.05;

** p < 0.001. Lower and Upper represent a 95% confidence interval for the mean difference from the midpoint.

To determine whether lay beliefs might account for the emotions experienced as a result of mindwandering, we computed the correlation between lay beliefs about the causes of mindwandering and anticipated positive and negative affect after a mindwandering event. As expected, greater endorsement of the belief that mindwandering is caused by a distractible disposition is associated with anticipations of less positive and more negative feelings after off-task moments. Similarly, believing that mindwandering is caused by dull activities is associated with anticipations of negative feelings after off-task moments. In contrast, believing that mindwandering is the result of a natural waxing and waning of attention is associated with less anticipated negativity after an off-task moment (Table 2).

**Table 2:** **Prediction of mood subsequent to mindwandering, by Lay Beliefs**.

	**Correlation**	
**Most of my mindwandering**	**Positive affect**	**Negative affect**
Occurs because I'm a distractible person	−0.17^**^	0.13^*^
Is caused by dull activities	−0.30^**^	−0.08
Is the result of my attention waxing and waning naturally	−0.11^*^	−0.15^**^
Occurs because I enjoy thinking about the topics to which my mind wanders	0.02	−0.09
Occurs because the topics to which my mind wanders are important	−0.03	0.02
Occurs because the topics to which my mind wanders are worrisome	−0.02	0.15^**^

Respondents (N = 361) rated the degree to which they would feel positive and negative affect using the PANAS (Tellegen et al., 1988; 1 = very slightly or not at all; 5 = Extremely). Correlation between lay beliefs about the causes of mindwandering and anticipated moods after a hypothetical mindwandering event.

* p < 0.05;

** p < 0.01.

These results raise the possibility that beliefs about mindwandering can increase the likelihood that one will interpret (possibly erroneously) an off-moment thought as evidence of discontentment. It is important for us to emphasize that this is not evidence that people who endorse the view that mindwandering causes negative moods are wrong; not all lay beliefs are de facto incorrect. However, these data confirm that the affective experiences people report as a result of mindwandering are not independent of their explicit beliefs about when and why the mind leaves a current task.

## General discussion

In this piece we have surveyed the link between mindwandering and affect while sounding a note of caution about interpreting these results in a causal light. The observed connections between mindwandering and diminished well-being are complex and likely multiply determined. In particular, we suggest that there could be many “third factors” that could explain the association between mindwandering and diminished well-being. These factors include issues pertaining to individual dispositions (depression, mental self-regulation), life events (unresolved personal concerns, disconcerting news), and task characteristics (invariable or slow tasks).

In addition, we report data from two studies that speak to the potential challenges of establishing a definitive causal link between mindwandering and well-being. First, we advanced the idea that mindwandering can convey affective benefits, in spite of negative feelings about mental disengagement. Using fMRI in a unique individual, we found (1) an association between periods of active mindwandering and increased activity in reward areas of the brain and (2) spontaneous correlations during rest between important default network areas and these same reward regions. By no means do we seek to imply that all mindwandering experiences are pleasurable. Our aim was to highlight that the affect experienced while mindwandering and the affective reactions to identifying a mindwandering episode are dissociable. We presented evidence of this in an individual being treated for a daydreaming compulsion (Schupak and Rosenthal, 2009). Whether the findings generalize beyond this particular person is certainly an open question. However, we suspect she is not unique in her ability to enjoy her off task-moments despite a concern about their occurrence.

We also probed the idea that people's beliefs (lay theories) about mindwandering may substantially color the affective responses they anticipate after a mindwandering episode (Study 2). Using a survey study, we showed that participants who endorsed negative lay beliefs about mindwandering also anticipated negative affective reactions following a mindwandering episode. Likewise, people who believe periodic disengagement from the external environment is a natural feature of our information processing system anticipated less negativity following a mindwandering episode than people who don't endorse this causal account of mindwandering. Importantly, Study 2 falls short of demonstrating that people who hold a lay belief that mindwandering causes negative moods are incorrect. Future research is required to examine this possibility in greater detail. Still, these results indicate a need to take account of lay theories of mindwandering when interpreting the affective data reported following a mindwandering event.

We conclude that the relationship between mindwandering and affect is complex and that far greater specification is needed before firm conclusions can be drawn regarding the causal processes at play. In lieu of such evidence, it seems appropriate to refrain from assuming our capacity for decoupling attention to ongoing tasks and events necessarily diminishes our happiness. Future research might examine the conditions under which this decoupling ability poses a risk to our emotional wellbeing and when it confers emotional benefits.

### Conflict of interest statement

The authors declare that the research was conducted in the absence of any commercial or financial relationships that could be construed as a potential conflict of interest.

## Appendix

### Affective reactions to mindwandering: fMRI study

#### Method

***Participant.*** The participant is a professionally accomplished 40-year-old female presenting with a long history of excessive and highly structured daydreaming which she states has contributed to considerable distress during periods of her life, despite being a “treasured activity”. The patient does not smoke, drink, or use drugs and has no abuse or trauma in her history. More details of her situation can be found in the case report written by Schupak and Rosenthal (2009).

***Procedure.*** The participant performed three different tasks. She was instructed to let her mind wander freely when prompted with the word “daydream” (i.e., during the mindwandering blocks), to actively prevent her mind from wandering when prompted with the word “control” (i.e., during the mindwandering suppression blocks), and to indicate whether the word on the screen was abstract or concrete during the “concentrate” blocks (Pagnoni et al., 2008; Andrews-Hanna et al., 2010). To obtain whole brain images quickly, we utilized echo-planar imaging (EPI) technology. Each of the two EPI series consisted of six mindwandering blocks (each lasted 60–80 s. in duration), three blocks of active mindwandering prevention (each lasted 30 s. in duration), and three blocks of the lexical decision task (each lasted 30 s. in duration). Ten seconds of fixation were interspersed between task blocks.

The stimuli were presented using Presentation (version 12.1) and back projected with an LCD projector onto a screen at the end of a magnet bore, which the participant viewed by way of a mirror mounted on the head coil. We placed pillow and foam cushions within the head coil to minimize head movements.

***Image acquisition.*** We collected all images using a GE scanner with a standard head coil. We collected T1-weighted anatomical images using a 3-D sequence (SPGR; 180 axial slices, TR = 19 ms, TE = 5 ms, flip angle = 20°, FOV = 25.6 cm, slice thickness = 1 mm, matrix = 256 × 256), and collected functional images with a gradient echo EPI sequence (each volume comprised 27 slices, 4 mm thick, 0 mm skip; TR = 2 s, TE = 35 ms, FOV = 19.2 cm, 64 × 64 matrix; 84° flip angle). We collected a total of 720 image volumes across two runs that were each 12 min in duration.

***Data analysis.*** We analyzed fMRI data using Statistical Parametric Mapping software (SPM5, Welcome Department of Cognitive Neurology, London, UK). For each functional run, we preprocessed the data to remove sources of noise and artifact. Preprocessing included slice timing and motion correction, co-registration to the participant's anatomical data, normalization to the ICBM 152 brain template (Montreal Neurological Institute), and spatial smoothing with an 8 mm (full-width-at-half-maximum) Gaussian kernel.

A general linear model with 28 regressors was specified (Friston et al., 1994). The model included a regressor for each of the three block types (concentrate, mindwandering, mindwandering suppression; 6 total), a regressor for each of the first four volumes collected in the two EPI series (8 total), six motion-related regressors for each series (12 total), and the two SPM constant terms. We used the general linear model to compute parameter estimates (β) and *t*-contrast images for each comparison at each voxel.

#### Results

Results of a direct contrast revealed significantly greater activity in both bilateral dorsal (peak MNI = 24, 16, −14; *t*_(670)_ = 6.44, *p* < 0.001, uncorrected) and bilateral ventral (nACC; peak MNI = 10, 22, −10; *t*_(670)_ = 5.84, *p* < 0.0001, uncorrected) striatum during mindwandering blocks relative to concentration blocks. Results of a direct contrast also revealed significantly greater activity in bilateral dorsal striatum (peak MNI = −20, 12, 0; *t*_(670)_ = 4.07, *p* < 0.001, uncorrected) during mindwandering blocks relative to mindwandering suppression blocks. See Figure 1.

### Affective reactions to mindwandering: functional connectivity study

#### Materials and procedure

We instructed the participant to keep her eyes open and look at a centrally presented fixation cross (“+”).

#### Image acquisition

The T1 and T2 imaging parameters used in the functional imaging study were used here. We collected a total of 360 image volumes across two runs that were each 6 min in duration.

#### Preprocessing

We carried out image preprocessing using FSL (Woolrich et al., 2009). Anatomical images were skull stripped using BET and partial volume estimates for gray matter (GM), white matter (WM), and cerebrospinal fluid/non-brain tissue (CSF) obtained using FLS's FAST. All EPIs were slice time corrected, realigned to the first image, and unwarped to correct for movement artifacts (Andersson et al., 2009). The T1-weighted structural image was coregistered to the mean EPI image and spatially normalized to the Montreal Neurological Institute (MNI) T1-weighted template in FSL, resulting in 2 mm × 2 mm × 2 mm voxels. EPI images were high pass filtered (100 s cutoff) and spatially smoothed using an isotropic Gaussian filter (6 mm FWHM). We preprocessed both resting-state sessions identically. Before connectivity analysis, we dropped the first 20 images to remove transients.

#### Seed selection

We defined a seed region based on the participant's anatomical data. We defined the seed signal as the average signal in a sphere of a 10 mm radius centered on the nACC seed (MNI coordinates: 14, 10, −6).

#### Functional connectivity analysis

A good general introduction to this type of analysis is given elsewhere (Fox et al., 2005); we describe our own implementation here. We selected all brain voxels using the extracted, whole-brain normalized anatomical image. We considered voxels in this image passing an intensity threshold of 0.1 (maximum intensity 1.0) as belonging to the brain. We used partial volume images produced from FAST to compute detrended mean WM and CSF images; we used any voxel with intensity greater than 0.5 in the WM and CSF images in constructing the mean WM and CSF signals, respectively. Before mean seed signal computation, we regressed all six motion parameters, mean WM and mean CSF, and their first derivatives (16 time series) out of the brain data. We then used these residual images in seed-signal calculation (described above) and subsequent functional correlation. Before calculation of seed-voxel correlations, we resampled all signals to remove the effects of serial autocorrelation. We then constructed correlation images for both resting state sessions. For each correlation computed, we used a simple bootstrap to estimate confidence limits (Chernick, 1999). We bootstrapped each correlation 200 times and computed 95% confidence intervals using the bootstrap distribution. If a correlation's 95% CI overlapped zero, we deemed the correlation insignificant. Doing so produced an image for each resting state session; we displayed correlations in Figure 2 only if they were significantly different from zero in *both* resting state sessions.

### Lay theories of mindwandering

#### Method

***Participants.*** A total of 378 individuals recruited via Amazon's Mechanical Turk marketplace completed the study. We excluded 17 participants from the analysis for failing to provide an accurate response to either of two items that we included as a quality control measure.

***Procedure.*** Participants were told that we were interested in how they would feel in a hypothetical situation. They were told that, although they would not have much information about the situation, they should answer the questions that followed to the best of their ability. Each participant was then prompted to imagine that we asked him or her to perform a task for about 4–5 min and that, 2-min and 15 s into performing the task, they noticed their mind had wandered to another topic. That is, they caught themself thinking about something that was unrelated to the ongoing task. Participants were asked to indicate the extent to which the following would characterize their mood at that precise moment in time (i.e., just after noticing that your mind wandered) on a scale from 1 = very slightly or not at all to 5 = extremely (Tellegen et al., 1988; PANAS).

After participants reported their anticipated post-wandering mood, they were asked to indicate the extent to which they agreed with various statements about the causes of mindwandering. More specifically, that it is caused by (1) dull tasks (To what extent do you think your mindwandering mostly occurs because of dull or monotonous tasks/events? To what extent do you think the reason for most of your mindwandering has nothing to do with the dullness of ongoing tasks or events?); (2) natural state of the mind (To what extent do you think most of your mindwandering occurs because attention tends to wax and wane naturally? In other words, most of it occurs because the mind tends to periodically disengage from ongoing events and activities? To what extent do you think the reason for most of your mindwandering has nothing to do with the mind's tendency to periodically disengage from ongoing events and activities?); (3) important topics (To what extent do you think most of your mindwandering occurs because important topics tend to capture your attention? To what extent do you think most of the reason for your mindwandering has nothing to do with the tendency for important topics capturing your attention?); (4) distractible dispositions (To what extent do you think most of your mindwandering occurs because you are a distractible person? To what extent do you think the reason for most of your mindwandering has nothing to do with how distractible you are?); (5) worrisome topics (To what extent do you think most of your mindwandering occurs because you are worried about the topics that capture your attention? To what extent do you think the reason for most of your mindwandering has *nothing* to do with being worried about the topics that capture your attention?); and (6) enjoyable topics (To what extent do you think most of your mindwandering occurs because you enjoy thinking about the topics that capture your attention? To what extent do you think the reason for most of your mindwandering had *nothing* to do with enjoying thinking about the topics that capture your attention?).

Finally, participants were asked to report their age, gender, and ethnicity.
